# Gastrointestinal transit measurements in mice with ^99m^Tc-DTPA-labeled activated charcoal using NanoSPECT-CT

**DOI:** 10.1186/2191-219X-3-60

**Published:** 2013-08-02

**Authors:** Parasuraman Padmanabhan, Johannes Grosse, Abu Bakar Md Ali Asad, George K Radda, Xavier Golay

**Affiliations:** 1 Laboratory of Molecular Imaging, Singapore Bioimaging Consortium (SBIC), A*STAR, 11 Biopolis way, Singapore 138667, Singapore; 2 Takeda Cambridge Ltd., 418 Cambridge Science Park, Cambridge CB4 0PA, UK; 3 UCL Institute of Neurology, Queen Square, London WC1N 3BG, UK; 4 The Lee Kong Chian School of Medicine, Nanyang Technological University, 50 Nanyang Drive, Singapore 637553, Singapore

**Keywords:** Tc-Ch-DTPA, SPECT-CT, GI transit, Loperamide, Bowel disorder

## Abstract

**Background:**

Gastrointestinal (GI) disorders are commonly associated with chronic conditions such as diabetes, obesity, and hypertension. Direct consequences are obstipation or diarrhea as opposite aspects of the irritable bowel syndrome, and more indirectly, alteration of appetite, feeling of fullness, flatulence, bloatedness, and eventually leading to altered absorption of nutrients. Moreover, GI retention and passage times have been recognized as important factors in determining the release site and hence the bioavailability of orally administered drugs. To facilitate the understanding of physiological and pathological processes involved, it is necessary to monitor the gut motility in animal models. Here, we describe a method for studying the GI transit time using technetium-labeled activated charcoal diethylenetriaminepentaacetic acid (^99m^Tc-Ch-DTPA) detected by single-photon emission computed tomography (SPECT).

**Methods:**

Tc-DTPA was adsorbed onto activated charcoal and administered orally to trypan blue-tainted (*n* = 4) 129SvEv mice (50 to 80 MBq/animal, *n* = 11). The exact distribution and movement of radioactivity in the gastrointestinal tract was measured at intervals of 1, 3, 6, 12, and 22 h by SPECT-CT. In addition, in order to validate the imaging of GI transient time, loperamide (0.25 mg/animal, *n* = 3) was used to delay the GI transit.

**Results:**

The transit time measured as the peak radioactivity occurring in the rectum was 6 to 7 h after gavaging of ^99m^Tc-Ch-DTPA. After 1 h, the bolus had passed into the small intestine and entered the cecum and the colon. At 6 and 8 h, the cecum, the ascending, transverse, and descending colon, and the rectum showed significant labeling. Several pellets were stored in the rectum for defecation. After 22 h, little activity remained in the stomach and none was detected in the transverse colon or other GI locations. In contrast, 6 h after administration of loperamide, only the cecum and part of the transverse colon were labeled. After 22 h, both structures retained significant amount of label. This delay has been verified by non-radiolabeled dye trypan blue GI measurements (*n* = 4).

**Conclusion:**

Here, we present the first non-invasive study of mouse GI transit time, allowing clear differentiation between vehicle- and loperamide-treated animals. This technique is useful for the investigation of GI motility in mice.

## Background

Measurement of gastrointestinal (GI) transit time is a very useful clinical and research technique for evaluating the motility disorders in the gastrointestinal tract. A number of testing methods are available and used frequently by the clinicians to evaluate the symptoms and causes of the GI transit, motility, and drug release in humans. These measurements will provide an assessment of the overall motility. The abnormality in gastrointestinal transit is considered as an unfathomable symptom. Normally, symptoms appearing in one part of the gut may overlap with symptoms from another; so, using direct *in vivo* measurements in transit studies is an important tool for diagnostic evaluations [1].

A variety of methods have been used for assessing GI transit times, motility, and drug release. Most well known is the use of X-ray [2] and scintigraphic techniques [3] which have been used to observe orally ingested capsules containing radiopaque substances or gamma emitters, respectively. On the other hand, non-invasive techniques such as ultrasound [4], metal detectors [5], magnetic field detectors [6], and dyes [7] have been used to avoid the adverse effects of ionizing radiation. However, all these methods have limitations due to fundamental constraints such as low temporal or spatial resolution, lack of balancing anatomical information, or incomplete spatial information. Furthermore, such capsules being foreign in nature and having considerably large size may not reflect an accurate physiology of the GI tract.

Despite the considerable efforts undertaken by researchers in academia and industry alike during the past two decades, the success rate for effective drug development for irritable bowel syndrome (IBS) and other functional gastrointestinal disorders did not score satisfactorily [8]. GI motility and functional disorders are interrelated and are usually expressed as undefined symptoms, either alone or overlap with each other. These are presented as two distinct categories; the first encompasses swallowing disorder, diarrhea, vomiting, and abnormal pain such as functional dyspepsia and IBS. The second category refers to esophageal, gastric, intestinal, and colonic motility and chronic idiopathic intestinal pseudo-obstruction.

Current *in vivo* methods are mainly based on single time point measurements for the small intestine, using either charcoal or similar inert non-reabsorbable dyes. Thus far, imaging of radiotracers has been used in humans but not in small rodents. The large intestine is especially difficult to assess in mice. The only useful method is the total GI tract transit time measured using a tracer passage. The present study establishes a protocol for the measurement of gastrointestinal motility in small animals. The technique described in this study, to our knowledge, is the first method to measure longitudinal gastrointestinal motility in mice and would be useful for the study of pharmacological compounds, gastrointestinal hormones, and for transgenic animal models with altered GI motility.

## Methods

In this study, we used two contrasting materials for the labeling study in order to select the best reagent. A comparative study was carried out to examine the best preparation for the study; technetium-labeled activated charcoal diethylenetriaminepentaacetic acid (^99m^Tc-Ch-DTPA) was either used with activated charcoal or mixed with resin directly.

### Preparation of ^99m^Tc-Amberlite resin for gastrointestinal transit

Indigestible Amberlite resin pellets (approximately 1 mm diameter; Sigma-Aldrich, St. Louis, MO, USA) were radiolabeled with the radioisotope technetium ^99m^Tc, following the method of Theodorakis [9]. Briefly, 2.0 g resin was stirred in a vial with a solution of ^99m^Tc-sodium pertechnetate (400 to 500 MBq) in 2 ml saline. The mixture was stirred for 10 min, and the labeled resin was recovered by filtration (0.22 μm, Millipore, Billerica, MA, USA), washed, and then resuspended in distilled water. The fluid formulation used was a non-absorbable aqueous solution of ^99m^Tc-labeled Amberlite resin in saline.

### Preparation of charcoal-^99m^Tc-DTPA for gastrointestinal transit

To obtain ^99m^Tc-diethylene-triamine-pentaacetic acid (^99m^Tc-DTPA), a reducing agent is required, usually tin ion as Sn(OH)_2_. It is important to do all the mixing in an anaerobic atmosphere, e.g., nitrogen. In the preformulated kits, each vial contains 35 mg of DTPA and 2 mg of stannous chloride dihydrate (SnCl·2H_2_O) in freeze-dried form in a nitrogen atmosphere and the pH is adjusted to 4. It can bind 0.1 to 6 GBq of ^99m^Tc. Five-milligram DTPA was dissolved in ultrapure water (2 to 4 ml, the amount depends on how many animals are to be gavaged) under nitrogen atmosphere. Dissolution is facilitated by alkalinization (add 200 to 300 μL of 1 N NaOH). After dissolving DTPA, 0.3 mg Sn(OH)_2_ is added (1 to 2 crystals). After the solution is adjusted to pH 7, 500 to 1,000 MBq pertechnetate is added followed by incubation for 5 min in normal atmosphere. Activated charcoal (50 mg) was mixed with the ^99m^Tc-DTPA. After 2-min mixing, the charcoal suspension was ready for administration.

### SPECT imaging

Single-photon emission computed tomography (SPECT) imaging was performed with four-headed multiplexing multipinhole NanoSPECT (Bioscan Inc., Washington D.C., USA). Each head was fitted with an application-specific tungsten collimator with nine pinholes. For this study, we used a mice aperture (aperture 3), which comprises a total of 36 individual 1-mm diameter pinholes (nine pinholes in every collimator, 4 × 9 = 36) providing a maximum resolution of 0.75 mm for SPECT imaging. The NanoSPECT was calibrated with a phantom, approximately the size of the animals, filled with known amount of ^99m^Tc. The axial FOV is extended using a step-and-shoot helical scan of the animal, with the user defining a range from 24 to 270 mm according to the region to be imaged. The energy peak for the camera was set at 140 keV. The mice were scanned at various time points with an initial activity of approximately 50 to 80 MBq ^99m^Tc-Ch-DTPA. An acquisition time of 30 s per view was chosen, resulting in acquisition times ranging from 30 to 40 min per animal. The 9-min CT imaging was performed immediately following the whole-body SPECT imaging with 50-μm resolution. Reconstruction of the images was performed without including attenuation correction. Reconstructed data from SPECT and CT were co-registered using InVivoScope (Bioscan Inc.) for further analysis and interpretation.

### Performance of transit studies in mice

All animal experiments were performed under an Institutional Animal Care and Use Committee (IACUC)-approved protocol from Takeda Cambridge, Singapore, and an IACUC-approved imaging service protocol of Singapore Bioimaging Consortium. Eleven healthy mice ranging in age between 8 to 12 weeks were used in this study. The charcoal was gavaged as 0.3 mL of ^99m^Tc-Ch-DTPA suspension using oral feeding syringe at time 0. After gavaging of the ^99m^Tc-Ch-DTPA, the animals were imaged in the NanoSPECT-CT under light inhalation anesthesia using isoflurane (1% isoflurane in 100% oxygen at a flow rate of 1 L/min). Due to short duration of the anesthesia and short recovery time, a major effect of anesthesia on GI motility is not expected. To visualize the path traveled by the charcoal along the total length of the gastrointestinal tract, temporal images were collected for post-acquisition analysis. SPECT scans were obtained at 0, 1, 3, 6, and 22 h after ingestion of the ^99m^Tc-Ch-DTPA. The GIT (gastrointestinal transit) was determined by identifying the leading front of intragastrically administered charcoal-labeled radiotracer in the GI tract. The GIT was calculated using the ^99m^Tc-Ch-DTPA movements on the basis of the activity residing in various locations within the GI tract. The total radiotracer activity at a given time point was calculated using the half-life coefficient with built-in HiSPECT software (Bioscan Inc.).

### Drug treatment

Two sets of male 129SvEv animals (3 to 5 months old, body weight 25 to 30 g) were used for the drug study: control- and loperamide-treated groups. The charcoal was mixed with the ^99m^Tc-DTPA and gavaged 30 min after loperamide administration (0.25 mg per animal via gavage) [10] in the drug study group and vehicle in control group. The loperamide dose was selected based on published data. The SPECT scans were obtained at 0, 1, 3, 6, and 22 h after ingestion of the ^99m^Tc-Ch-DTPA. Once the drug was given to the animals, they were allowed to reside in separate cages with *ad libitum* access to food and water. The animals were imaged at specified time points, and data were used for post-acquisition analysis.

### GIT measurement using trypan blue dye

Four healthy mice ranging in age between 8 and 12 weeks were used in this study; the charcoal (2.0 g) was mixed with 0.4% trypan blue and gavaged (0.3 ml) with the aid of an oral feeding syringe. Animals were sacrificed at varying time points viz. 1, 3, 6, and 22. At the first time point, the abdomen was opened and the stomach was examined at the cardiac and pyloric ends. This correlates with the concentration of trypan blue in the stomach. The other time points were the same as those used for the ^99m^Tc-Ch-DTPA measurements. The small intestine, cecum, transverse colon and large intestine loop, and colon region of trypan blue traveling time were recorded and compared with the SPECT images of ^99m^Tc-Ch-DTPA. The total travel time of GIT was determined on the basis of the excretion of first fecal pellet tainted with trypan blue. The color of the pellets was compared with those from the start of the experiment.

### Quantification of the activity at different transit times

After the acquisition, the data were reconstructed iteratively with the HiSPECT software (Bioscan Inc.) using dedicated ordered subsets expectation maximization for multiplexing multipinhole reconstruction. A region of interest (ROI) was drawn manually around the transit portion of the GI tract; the 3D activity distribution within the ROI was then summed to determine the uptake on the basis of each frame. Scatter correction was not performed. All measured activities were corrected for decay and expressed as percent injected (gavage) activity (%IA). The ingested activity was determined by measuring the syringe in a dose calibrator (Biodex Atomlab, New York, NY, USA) before and after the oral gavage of the animal. The difference was defined as the injected activity. Quantification of the ROI is performed with the InterView XP software (Mediso Ltd., Budapest, Hungary).

### Histological staining

Gastric specimens of normal charcoal gavaged without radioactive label and charcoal gavaged labeled with ^99m^Tc-DTPA were fixed in 10% neutral-buffered formalin. The paraffin-embedded tissues were sectioned and stained with hematoxylin and eosin for standard histological evaluation. The stained sections were photographed using a 90I Nikon microscope with FL/DIC/Phase (Melville, NY, USA).

## Results

**Figure 1:**
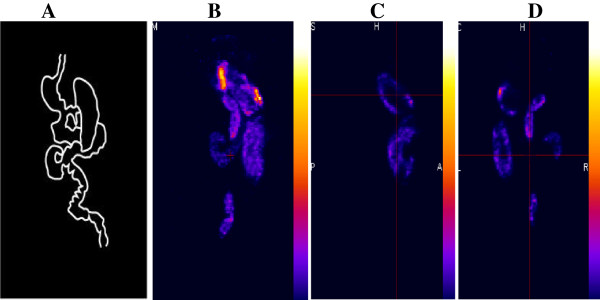
**Movements of **^**99m**^**Tc-Ch-DTPA in the GI tract after 7 h of gavaging. (A)** Scheme of mouse GI tract. **(B)** MIP image of SPECT after 7 h. **(C)** Sagittal image of SPECT after 7 h. **(D)** Coronal image of SPECT after 7 h.

**Figure 2:**
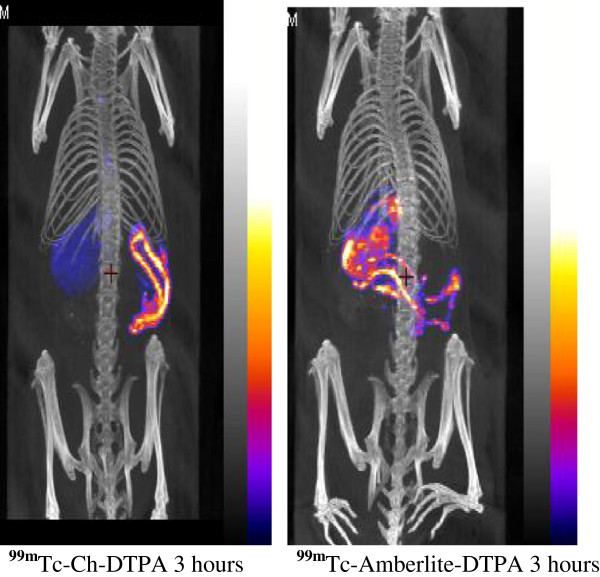
**Comparison between **^**99m**^**Tc-Ch-DTPA and **^**99m**^**Tc-Amberlite resin movements in the GI tract after 3 h of gavaging.**

The aim of this study was to develop a novel non-invasive *in vivo* method for quantifying the gastrointestinal transit in live animals (Figure 1). The conventional methods of using dye-based transit studies provide only single time points; thus, longitudinal studies are not possible. In our study, we used two sets of materials for labeling, in order to select the best reagent for these experiments. Firstly, we labeled the activated charcoal with ^99m^Tc-DTPA, and in another, unconjugated ^99m^Tc was mixed with Amberlite resin. The images collected at 3-h time point of both gavaged animals showed significant gastrointestinal labeling, but the charcoal gavaged showed better image than the Amberlite resin (Figure 2). Although earlier studies, in common brushtail possum and in dog using gamma scintigraphy, have used ^99m^Tc-labeled resin for GI motility measurements [11,12], these proved to be less sensitive than our NanoSPECT imaging. Our present study was more sensitive in terms of binding affinity and measurements with charcoal compared to ^99m^Tc-labeled resin method.

**Figure 3:**
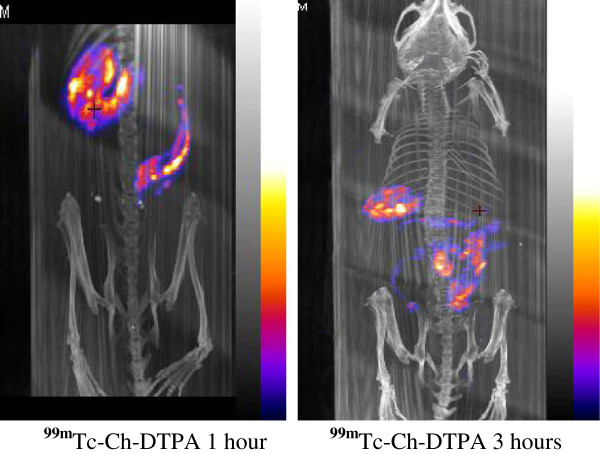
^**99m**^**Tc-Ch-DTPA movements in the GI tract after 1 and 3 h of gavaging.**

The images collected at different time points were processed for extracting different information. It was noticed that 1 h following gavaging, the total charcoal bolus had entered the stomach and begun entering into the small intestine. After 3-h transit time, the ^99m^Tc-Ch-DTPA had labeled almost the entire loop of small intestines and cecum (Figure 3).

**Figure 4:**
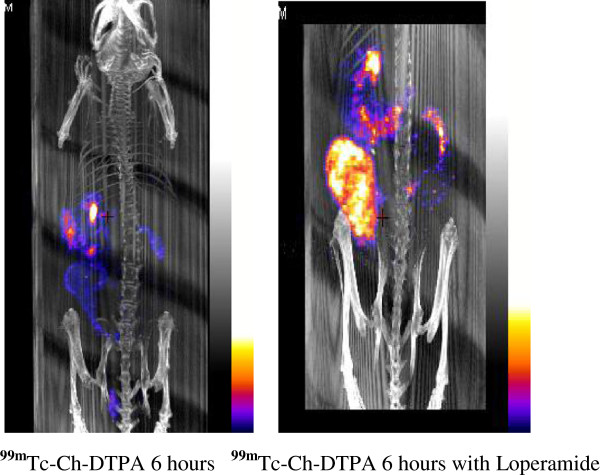
**After 6-h gavaging of **^**99m**^**Tc-Ch-DTPA showing the movements in the GI tract with and without loperamide.**

**Figure 5:**
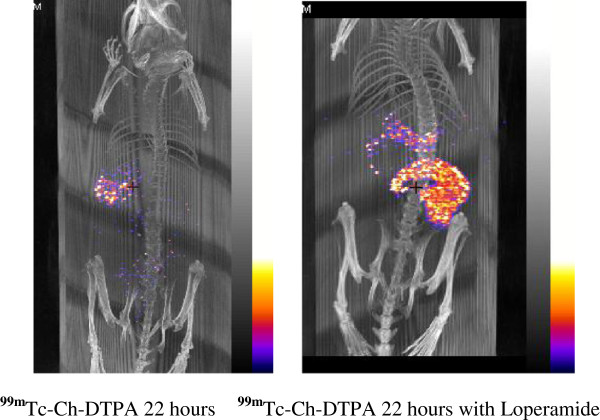
**Movements of **^**99m**^**Tc-Ch-DTPA in the GI tract after 22 h of gavaging with and without loperamide.**

**Figure 6:**
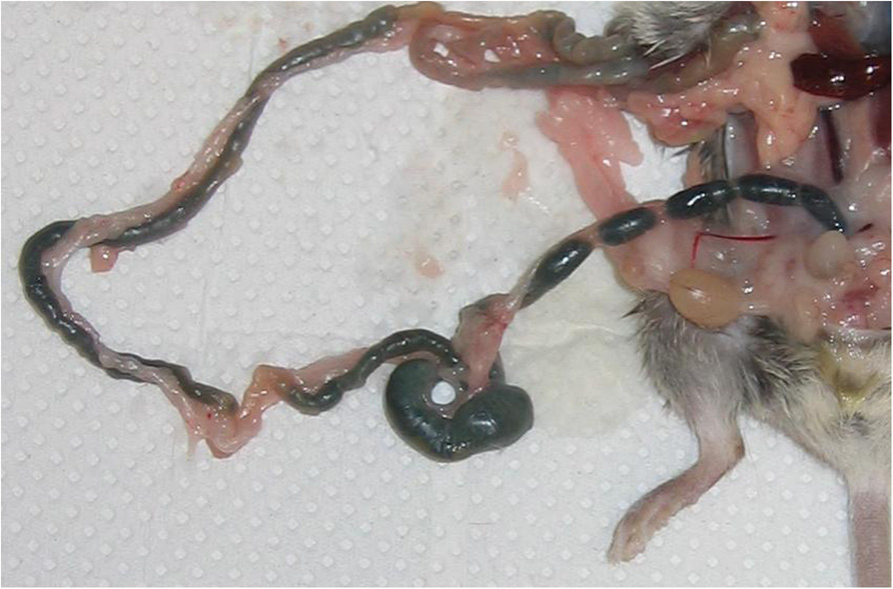
**Trypan blue-gavaged animal after 6 h that resembles a similar total transit time observed with **^**99m**^**Tc-Ch-DTPA.**

After 6 and 8 h, the distinct labeling of ^99m^Tc-Ch-DTPA was visualized in the cecum, the ascending, transverse, and descending colon, and the rectum (Table 1). Several pellets were stored in the rectum before defecation. We conclude that the GI transit time is approximately 6 h (Figure 4, left). After 22 h following the gavage, only little radioactivity remained in the stomach (Figure 5, left) and nothing was seen in the transverse colon or other location along the GI tract (Table 2). Furthermore, we confirmed the same total transit time by gavaging trypan blue, a non-radioactive dye, and dissecting the animals open at different time points to observe the GI motility. This experiment employing trypan blue also further confirms that there is no underlying toxicity that may potentially impair the GI physiology and integrity due to the use of radioligand in the GI tract. This was confirmed by sacrificing the animal at every imaging time point. Figure 6 shows an animal sacrificed 6 h after trypan blue gavage. The post-mortem analysis of animals at different time points to observe the GI motility demonstrated that there was no difference in the transit time between the two methods.

**Table 1:** Transit measurements in GI tract using charcoal Tc-DTPA at different time points

		
**Specimen number**	**After gavages of charcoal Tc-DTPA**	**Transit measurements in the GI tract**
1	1 h and 10 min	Stomach and small intestine are clearly labeled
2	3 h	Stomach and small intestines are clearly labeled; leading front is just entering into the cecum
3	6 h	Whole GI tract is prominently labeled; well-defined cecum, transverse colon and large intestinal loops, and prominent labeling of the colon were seen
4	8 h	The transverse colon, descending colon and fecal pellets in the rectum are clearly labeled
5	22 h	Only little activity remains in the stomach (potentially parietal cells accumulating free Tc)

**Table 2:** **Transit measurements in the GI tract using charcoal **^**99m**^**Tc-DTPA at different time points after administration of loperamide**

		
**Specimen number**	**After gavages of **^**99m**^**Tc-ChDTPA with loperamide**	**Transit measurements in the GI tract**
1	1 h	Stomach and small intestine are very clearly labeled
2	6 h	Stomach and well-defined labeling of the cecum is seen
3	22 h	Transverse colon and cecum are labeled

**Figure 7:**
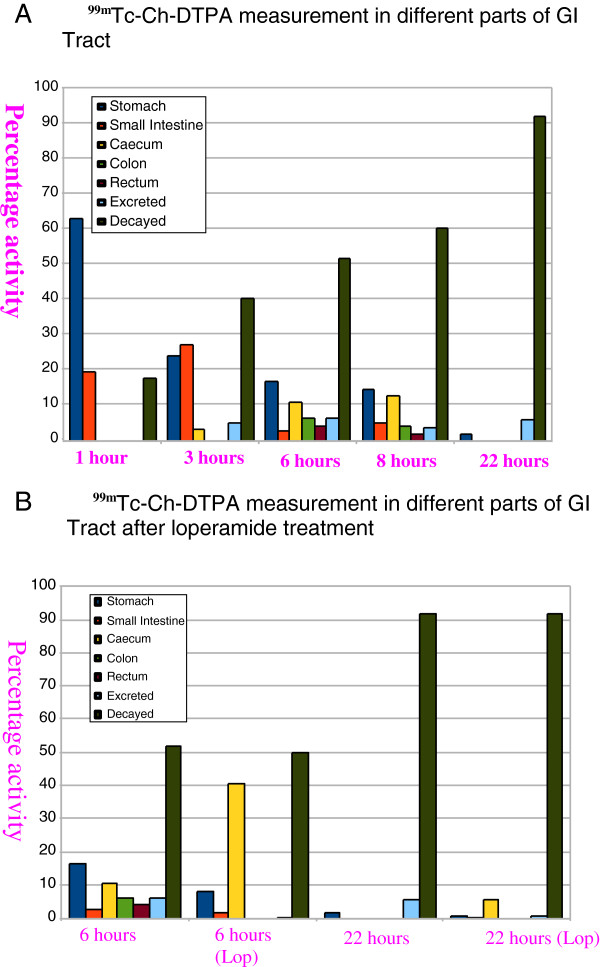
**Showing **^**99m**^**Tc-Ch-DTPA measurement in different parts of GI tract (A and B).** Before and after loperamide treatment.

To delineate the pharmacological modification of GI transit time, we selected the drug, loperamide, administered 30 min after ^99m^Tc-Ch-DTPA gavaging. Loperamide is known to delay the GI transit time in both animals and humans by activating μ-opioid receptors of the myenteric plexus. After 6 h of loperamide administration, the animals showed clear ^99m^Tc-Ch-DTPA labeling in stomach, cecum, and transverse colon (Figure 4, right). Even imaging 22 h post-gavage with SPECT showed that both the cecum and transverse colon retained significant label (Figure 5, right). To quantify the activity at different time points post-gavage, charcoal motility was analyzed and calculated, normalizing all parameters (Figure 7A, B). As expected, loperamide results in a slower progression of the radiotracer.

**Figure 8:**
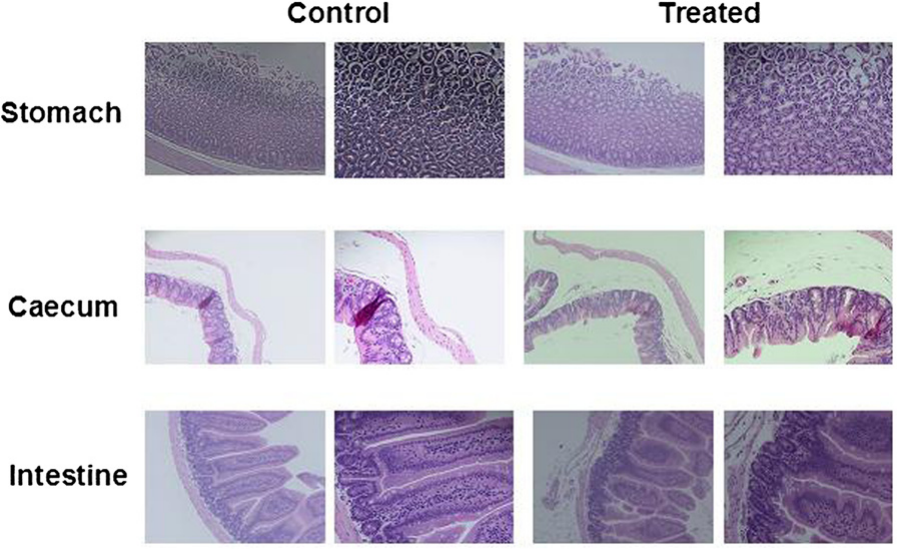
Light microscopy pictures of normal and Tc-Ch-DTPA-treated sections stained with HE.

In order to observe any toxicity of the labeled radiotracer (^99m^Tc-Ch-DTPA), we fixed the tissues after 5-h administration of ^99m^Tc-Ch-DTPA at the three regions of GI tract, namely the stomach, intestine, and colon for histology. The sections were compared with the normal tissues at the same locations. The histological sections of the above tissues showed no significant damage in the tissue, which suggests that there is no toxicity during the transit of the ^99m^Tc-Ch-DTPA through the GI tract (Figure 8).

## Discussion

The tracing of radiopharmaceuticals *in vivo* offers immense potential in order to understand their quantification and mechanism of action allowing the development and analysis of new drugs. Non-invasive *in vivo* imaging, with quantification methods of radioactive compounds, allows the determination of the accurate uptake of target molecules. In our study, we used ^99m^Tc-Ch-DTPA which is unique and efficient for the imaging of the GI tract motility. In our comparison with the ^99m^Tc-Amberlite resin and ^99m^Tc-Ch-DTPA for transit motility, the ^99m^Tc-Ch-DTPA was better retained compared with ^99m^Tc-Amberlite resin. The same type of scintigraphic measurement has been reported in humans using activated charcoal [13].

McDowell et al. [14] has reported that the measurements on gastrointestinal tracts of *Trichosurus vulpecula* in New Zealand are compared with those of the published information. Another aspect of the same study is that they have shown the inter-animal variation in different areas and the pH of the gastrointestinal tract of individual possums. So far, one transit time study has been reported with the fluid and indigestible particles to determine in the common brushtail possum (*T. vulpecula*). ^99m^Tc-labeled resin (anion) particles (sizes ranging from 75 to 125 or 500 to 700 μm diameters) or solution (^99m^Tc-DTPA) was administered orally. They predetermined the times after dosing (3, 6, 12, 24, or 32 h), the distribution of radioactivity measured *ex vivo* on different time points [12].

In the present study, the GI transit was assessed by the NanoSPECT using the ^99m^Tc-Ch-DTPA. The spatial resolution of 0.45 mm allowed the observation of the GI transit movement with excellent accuracy. We found that the total GI transit time is around 6 h in mice. Out of 11 animals scanned, at several time points 0, 1, 3, 6, 8, and 22 h, we found that the entire GI tract was cleared between 6 and 8 h. Our non-radiolabeled dye study also supported these findings (data not shown). The half-life of ^99m^Tc is 6 h; thus, the image obtained after 22 h in normal mice showed no activity in transverse colon and other location, with only trace amounts remaining in the stomach, which clearly indicates that the free ^99m^Tc was accumulated in the labeled parietal cells present in the stomach.

To validate the toxicity issue of the ^99m^Tc-Ch-DTPA, the present results, the histology has been obtained at different regions of the GI tract and there are no significant changes in all portions at different times of exposure. This clearly shows that the dose we used on the basis of animal weight is appropriate for the mice in all respects. There are reports on the toxicity of the radioactivity; it is mostly on different organs and not on the digestive tracts with technetium [15,16].

To test the delay in the transit time, we administered loperamide. The drug, loperamide, normally slows the muscular contractions of the intestine and is thus termed as an antimotility agent. Loperamide acts mainly on opioid receptors, which are found in the walls lining the intestines. This reduces the muscular contractions of the intestine (peristalsis) that move food and fecal matter through the gut. After 6 h of administration, the GI tract was labeled only at the stomach, cecum, and transverse colon. This clearly indicates that the drug has tremendous effect of delaying the transit time. Even 22 h later, the images show the retention of some labeling in the same structures, further supporting the mechanism of drug action.

## Conclusion

In summary, we present the first non-invasive radioactive-based monitoring of gastrointestinal transit time of mouse in real time, using NanoSPECT, allowing clear depiction of differences between drug-free and loperamide-treated animals. Furthermore, it demonstrates the feasibility of studying the site of release of pharmaceutical compounds *in vivo*.

## Competing interests

The authors declare that they have no competing interests.

## Authors’ contributions

PP, JG, and ABMAA designed the experiments, contributed to the experimental analysis and interpretation of data, and contributed to the manuscript preparation. PP and ABMAA wrote the manuscript and revised the manuscript from other co-authors. GKR designed the experiments, contributed to the interpretation of the experimental data, and edited the manuscript. XG conceived the work and contributed to the interpretation of the experimental data and editing the manuscript. All authors read and approved the final manuscript.

## References

[B1] Lin H Prather C Fisher R Meyer J Summers R Pimentel M McCallum R Akkermans L Loening-Baucke V Measurement of gastrointestinal transit Dig Dis Sci 2005 3 989 1004 15986844 10.1007/s10620-005-2694-6

[B2] Abrahamsson H Antov S Bosaeus I Gastrointestinal and colonic segmental transit time evaluated by a single abdominal X-ray in healthy subjects and constipated patients Scand J Gastroenterol 1988 3 72 80 10.3109/00365528809095938 3254616

[B3] Camilleri M Colemont LJ Phillips SF Brown ML Thomforde GM Chapman N Zinsmeister AR Human gastric emptying and colonic filling of solids characterized by a new method Am J Physiol Gastrointest Liver Physiol 1989 3 G284 290 10.1152/ajpgi.1989.257.2.G284 2764112

[B4] Amend M Greiner L The ultrasound capsule: a new method for measurement of gastrointestinal motility Ultraschall Med 1996 3 274 276 10.1055/s-2007-1003197 9082552

[B5] Ewe K Press AG Dederer W Gastrointestinal transit of undigestible solids measured by metal detector EAS II Eur J Clin Invest 1989 3 291 297 10.1111/j.1365-2362.1989.tb00232.x 2509213

[B6] Weitschies W Wedemeyer J Stehr R Trahms L Magnetic markers as a noninvasive tool to monitor gastrointestinal transit IEEE Trans Biomed Eng 1994 3 192 195 10.1109/10.284931 8026852

[B7] Leng-Peschlow E Effect of sennosides and related compounds on intestinal transit in the rat Pharmacology 1988 3 Suppl 1 40 48 3368525 10.1159/000138420

[B8] Mayer EA Bradesi S Chang L Spiegel BM Bueller JA Naliboff BD Functional GI disorders: from animal models to drug development Gut 2008 3 384 404 10.1136/gut.2006.101675 17965064 PMC4130737

[B9] Theodorakis MC External scintigraphy in measuring rate of gastric emptying in beagles Am J Physiol 1980 3 G39 43 7396001 10.1152/ajpgi.1980.239.1.G39

[B10] Tan-No K Niijima F Nakagawasai O Sato T Satoh S Tadano T Development of tolerance to the inhibitory effect of loperamide on gastrointestinal transit in mice Eur J Pharm Sci 2003 3 357 363 10.1016/j.ejps.2003.08.004 14592702

[B11] Iwanaga Y Wen J Thollander MS Kost LJ Thomforde GM Allen RG Phillips SF Scintigraphic measurement of regional gastrointestinal transit in the dog Am J Physiol 1998 3 G904 910 9815018 10.1152/ajpgi.1998.275.5.G904

[B12] McDowell A Nicoll JJ McLeod BJ Tucker IG Davies NM Gastrointestinal transit in the common brushtail possum measured by gamma scintigraphy Int J Pharm 2005 3 125 132 10.1016/j.ijpharm.2005.06.026 16112826

[B13] Burton DD Camilleri M Mullan BP Forstrom LA Hung JC Colonic transit scintigraphy labeled activated charcoal compared with ion exchange pellets J Nucl Med 1997 3 1807 1810 9374360

[B14] McDowell A McLeod B Thompson E Tucker I A morphometric study of the gastrointestinal tract of the common brushtail possum in southern New Zealand Aust Mamm 2005 3 61 67

[B15] Vera DR Stadalnik RC Krohn KA Technetium-99m galactosyl-neoglycoalbumin: preparation and preclinical studies J Nucl Med 1985 3 1157 1167 4045560

[B16] Dublineau I Grison S Baudelin C Dudoignon N Souidi M Marquette C Paquet F Aigueperse J Gourmelon P Absorption of uranium through the entire gastrointestinal tract of the rat Int J Radiat Biol 2005 3 473 482 10.1080/09553000500196029 16249162

